# Elucidation of the Transport Mechanism of Puerarin and Gastrodin and Their Interaction on the Absorption in a Caco-2 Cell Monolayer Model

**DOI:** 10.3390/molecules27041230

**Published:** 2022-02-11

**Authors:** Li Jiang, Yanling Xiong, Yu Tu, Wentong Zhang, Qiyun Zhang, Peng Nie, Xiaojun Yan, Hongning Liu, Ronghua Liu, Guoliang Xu

**Affiliations:** 1 Center for Differentiation and Development of TCM Basic Theory, Jiangxi University of Chinese Medicine, Nanchang 330004, China; jiangli1009@126.com (L.J.); t12532142@163.com (Y.T.); zhangzhang20504@163.com (W.Z.); 20060874@jxutcm.edu.cn (Q.Z.); 20091017@jxutcm.edu.cn (P.N.); 20040813@jxutcm.edu.cn (X.Y.); lhn0791@139.com (H.L.); 2 Jiangxi Provincial Key Laboratory of TCM Etiopathogenesis, Jiangxi University of Chinese Medicine, Nanchang 330004, China; 3 Key Laboratory of Pharmacology of Traditional Chinese Medicine in Jiangxi, Nanchang 330004, China; 4 Department of Clinical Pharmacology, Xiangya Hospital, Central South University, 87 Xiangya Road, Changsha 410008, China; xiongyanling1009@163.com; 5 Department of Pharmacy, Jiangxi University of Chinese Medicine, Nanchang 330004, China; 19890068@jxutcm.edu.cn

**Keywords:** transport mechanism, puerarin, gastrodin, absorption, Caco-2 cell monolayer

## Abstract

Puerarin (PUR) and gastrodin (GAS) are often used in combined way for treating diseases caused by microcirculation disorders. The current study aimed to investigate the absorption and transportation mechanism of PUR and GAS and their interaction via Caco-2 monolayer cell model. In this work, the concentration in Caco-2 cell of PUR and GAS was determined by HPLC method. The bidirectional transport of PUR and GAS and the inhibition of drug efflux including verapamil and cyclosporine on the transport of these two components were studied. The mutual influence between PUR and GAS, especially the effect of the latter on the former of the bidirectional transport were also investigated. The transport of 50 μg·mL^−1^ PUR in Caco-2 cells has no obvious directionality. While the transport of 100 and 200 μg·mL^−1^ PUR presents a strong directionality, and this directionality can be inhibited by verapamil and cyclosporine. When PUR and GAS were used in combination, GAS could increase the absorption of PUR while PUR had no obvious influence on GAS. Therefore, the compatibility of PUR and GAS is reasonable, and GAS can promote the transmembrane transport of PUR, the effect of which is similar to that of verapamil.

## 1. Introduction

**Figure 1:**
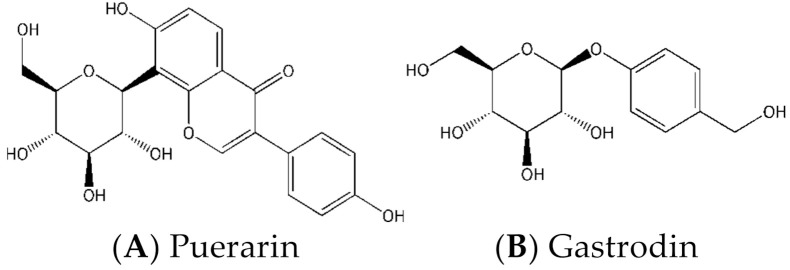
Chemical structures of Puerarin (PUR) and Gastrodin (GAS).

Puerarin (Figure 1A, PUR), chemically known as 8-*β*-d-glucopyranose-4′,7-dihydroxyisoflavone, is an important bioactive isoflavone glycoside, and it was isolated from several leguminous plants of the genus *Pueraria*, including *Pueraria tuberosa* (Willd.), *Pueraria lobata* (Willd.) Ohwi (Gegen in Chinese), and *Pueraria thomsonii* Benth [1,2,3]. PUR has the effects of dilating coronary arteries and cerebral blood vessels, improving microcirculation, anti-platelet aggregation, lowering blood pressure, and anti-oxidation [4]. It is mainly used to treat hypertension, coronary heart disease, angina pectoris, myocardial infarction, cerebral ischemia, migraine, sudden deafness and diabetes [5,6,7]. However, some of its physical properties, such as poor water solubility, poor permeability, and low oral bioavailability, lead to formulation of PUR is only for injection [8,9,10]. However, there are many reports of clinical adverse reactions caused by PUR injection, and the most serious is the hemolytic reaction, which is easily to cause the death of the patient when found and treated in an emergency. Therefore, there is widespread interest in how to improve the oral absorption of PUR. Gastrodin (Figure 1B, GAS) is an active component of *Gastrodia elata Blume*, chemically known as 4-hydroxybenzyl alcohol-4-*O*-*β*-d-glucopyranoside. Gas has a variety of pharmacological effects such as sedation and sleeping, promoting intelligence, protecting neurons, lowering blood pressure, antioxidant, and improving microcirculation, etc. It is extensively used to treat dizziness, migraine, high blood pressure, stroke and epilepsy [11,12,13,14,15].

In the treatment of cardiovascular and cerebrovascular diseases, PUR and GAS are often used in combination. In addition, many Chinese medicine prescriptions usually contain these two components [16,17]. In our previous study [18,19,20], the physicochemical properties (including solubility and oil-water partition coefficient), anti-oxidation effect on microcirculation (including DPPH free radical scavenging, anticoagulation and antiplatelet aggregation in vivo and in vitro) and pharmacokinetics in rats were analyzed to explore the compatibility rationality and interaction between PUR and GAS. The results showed that (1) GAS can increase the solubility of PUR, (2) anticoagulation and anti-platelet aggregation effects of PUR and GAS can be enhanced when used in combination in a certain range of dosage, and (3) the combined use of GAS can promote the absorption, decrease the elimination rate, and prolong the mean residence time of PUR in rats. However, the mechanism of GAS promoting the absorption of PUR is not interpreted. Thus, it is worth studying and exploring the promotion mechanism of PUR and GAS.

To our knowledge, there have been some studies on the intestinal absorption mechanism of PUR. With regard to GAS, for its good oral bioavailability, few studies were concerned to the absorption of GAS. Liang [21] found that PUR is absorbed by passive diffusion without energy consumption, and there is no obvious directionality in transportation. Moreover, Zhang [22] proposed that the reason for the poor uptake exhibited by PUR alone is mainly due to its mode of transport, which is a passive diffusion of the cell monolayer. Cheng et al. [23] found that adding verapamil could enhance the absorption of PUR, so it is believed that the transcellular transport of PUR was also mediated by P-glycoprotein (P-gp) in addition to passive diffusion.

The Caco-2 cell line was derived from human colon adenocarcinoma and its morphology and function are similar to those of human intestinal epithelial cells, and it contains enzymes related to the brush border epithelium of small intestine [24]. Caco-2 cell model has been widely used in the study of drug uptake, efflux, transmembrane transport and other absorption mechanisms at home and abroad in the past decade because of its good correlation, reproducibility and applicability with oral drug absorption in intestinal tract [25,26,27,28,29].

Thus, in this study, based on the clinical application and combination of PUR and GAS, Caco-2 cells was used to study the absorption and transportation mechanism of PUR and GAS and their mutual influence, so as to lay an experimental foundation for their feasibility of the compatibility.

## 2. Materials and Methods

### 2.1. Chemicals and Reagents

PUR and GAS were purchased from Wei Keqi Biotechnology Co., Ltd. (Chengdu, China) qualifying as administration drug and from National Institutes for food and drug control (Beijing, China), the purity >98%. P-hydroxy phenylethyl alcohol (internal standard, IS) was purchased from Aladdin reagent limited company (Shanghai, China), the purity >98%. Verapamil hydrochloride was obtained from National Institutes for food and drug control (Beijing, China). Cyclosporin was bought from Shanghai Jingke Chemical Technology Co., Ltd. (Shanghai, China). The Dulbecco’s modified Eagle’s medium (DMEM), and heat-inactivated fetal bovine serum (FBS) were obtained from Invitrogen. Penicillin (100 IU·mL^−1^) -streptomycin (100 μg·mL^−1^) double antidotic solution, trypsin, and dimethyl sulfoxide (DMSO) were purchased from Sigma Chemical Co. (St. Louis, MO, USA). Nonessential amino acids (NEAA) were obtained from Corning Cellgro (Manassas, VA, USA). Hank’s equilibrium salt solution (HBSS: 8 g·L^−1^ NaCl, 0.4 g·L^−1^ KCl, 1 g·L^−1^ glucose, 60 mg·L^−1^ KH_2_PO_4_, 47.5 mg·L^−1^ Na_2_HPO_4_, adjust pH to 7.4) was purchased from Beijing Solarbio Science and Technology Co., Ltd. (Beijing, China). Ethylene diamine tetraacetic acid (EDTA) was obtained from Merck and Co., Inc. (Kenilworth, NJ, USA). Acetonitrile was bought from Thermo Fisher Scientific (Beijing, China).

### 2.2. Cell Culture

Caco-2 cell line were initially obtained from the American Type Culture Collection (ATCC) and maintained in our laboratory. Caco-2 cells were maintained under humidity with 5% CO_2_ at 37 °C in DMEM supplemented 10% (*v*/*v*) heat-inactivated FBS, 1% non-essential amino acids, 1% L-glutamine, and penicillin (100 IU·mL^−1^)-streptomycin (100 μg·mL^−1^) double antidotic solution.

### 2.3. HPLC Analysis

#### 2.3.1. Instruments and Chromatographic Conditions

The analysis was performed using the Shimadzu HPLC system (Chiyoda-Ku, Kyoto, Japan). Analytes were separated on an Agilent ZORBAX SB-Aq C_18_ column (250 mm × 4.6 mm, 5 μm, Santa Clara, CA, USA) equipped with an Agilent analytical guard column (12.5 mm × 4.6 mm, 5 μm, Santa Clara, CA, USA).

The gradient mobile phase system, consisting of ACN (A)-H_2_O (B) and 0.05% phosphoric acid as modifier, was used to analyze the samples. The gradient elution method was as follows: 0–4.5 min: 3% A; 4.5–8 min: 3% A→15% A, 8–15 min: 15% A→30% A; 15–20 min: 30% A→30% A; 20–25 min: 3% A.

#### 2.3.2. Preparation of Working Solutions and Internal Standard (IS) Solution

10 mg PUR and GAS were precisely weighed and diluted with HBSS solution to obtain 1 mg·mL^−1^ PUR and GAS mixed solution, respectively. The above mixed solution was then diluted with HBSS solution to 0.08, 0.17, 0.33, 0.83, 1.67, 3.33, 8.33, 16.67, 25, 33.33, 50, 100, 166.67, and 333.33 μg·mL^−1^ working solution, respectively. Accurately weigh a certain amount of P-hydroxy phenylethyl alcohol (IS), and dilute to a concentration of 22.60 μg·mL^−1^ with HBSS solution.

#### 2.3.3. Preparation of Calibration Standard Samples

The calibration standard samples were prepared by transferring 30 μL of the each working solution into an Eppendorf tube (EP tube), to which 10 μL of IS and 60 μL methyl alcohol were added. The concentrations of calibration standard samples were 0.025, 0.05, 0.1, 0.25, 0.5, 1, 2.5, 5, 7.5, 10, 15, 30, 50, and 100 μg·mL^−1^, respectively.

#### 2.3.4. Preparation of Cell Samples

Transfer 30 μL of cell sample, 10 μL of p-hydroxy phenylethyl alcohol (IS) and 60 μL of methanol to an EP tube in turn, and vortex for 2 min. Then centrifuged at 12,000× *g*·min^−1^ for 10 min. Store the supernatant at 4 °C for subsequent analysis.

### 2.4. Method Validation

The specificity of the method was assessed by comparing the chromatograms of blank plasma, blank plasma spiked with internal standard/analyte, and cell samples. Calibration curves were established based on the peak area ratio versus nominal concentration. Intra-day and inter-day precision and accuracy are expressed by evaluating the measurement results of QC samples at 0.1, 5, and 100 μg·mL^−^^1^.

#### 2.4.1. Specificity

**Figure 2:**
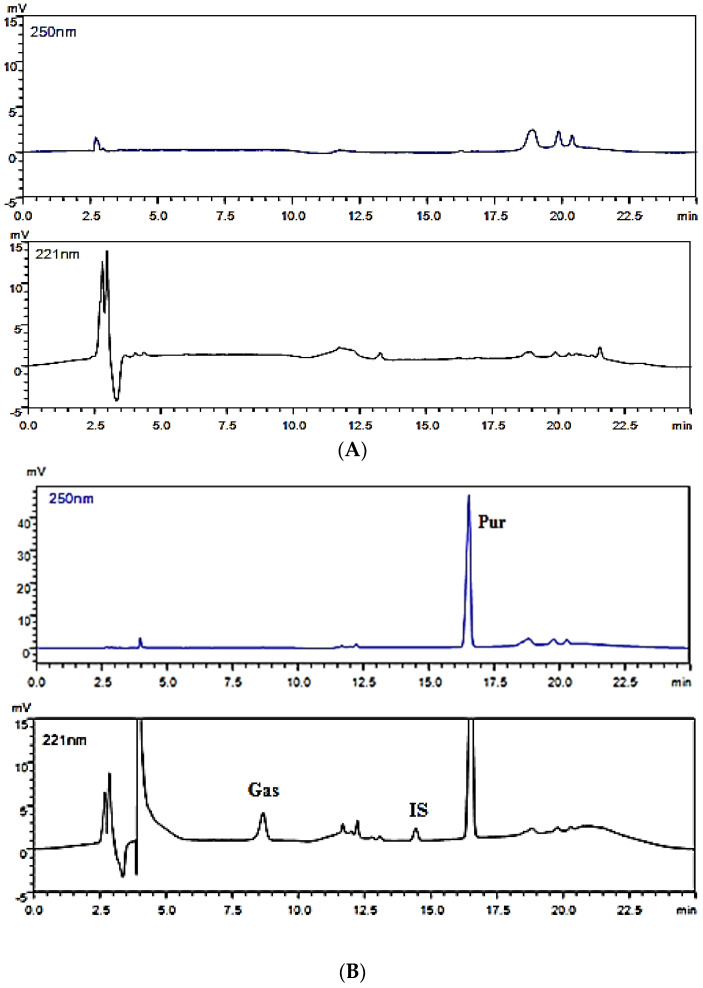

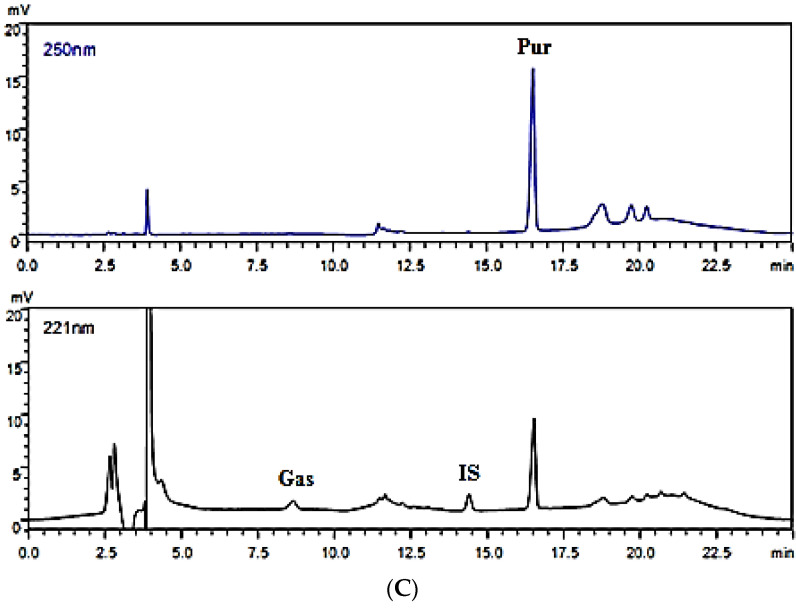
Typical HPLC chromatograms of puerarin (PUR), gastrodin (GAS), and internal standard (IS) in HBSS solution ((**A**): blank HBSS solution; (**B**): blank HBSS solution spiked with PUR, GAS and IS; (**C**): sample collected at 180 min after transportation of PUR and GAS from (**B**) to (**A**) side through Caco-2 cell).

The retention time for PUR, GAS, and p-hydroxy phenylethyl alcohol (IS) were 16.5, 8.6, and 14.8 min, respectively, and there were little interferential substances with the analytes and IS in the HBSS solution and cells. Representative chromatogram of analytes and IS in Caco-2 cell was shown in Figure 2.

#### 2.4.2. Linearity

Due to the wide range of concentration span, two standard curves of the analytes are made. For PUR, the low linear rang is 0.025–2.5 μg·mL^−1^, and the high linear rang is 2.5–100 μg·mL^−1^. For GAS, the low linear rang is 0.1–5 μg·mL^−1^, and the high linear rang is 5–100 μg·mL^−1^. The calibration curves of PUR and GAS exhibited good linearity, and the regression equations with correlation coefficients and linear range were listed in Table 1.

#### 2.4.3. Precision

Transfer 30 μL of each working solutions, and the samples were prepared in the same way as the cell samples at concentration of 0.1, 5, 100 μg·mL^−1^. Each of the six samples were processed in parallel. Precisions were expressed by the relative standard deviation (RSD, %). The intra-day and inter-day precision R.S.D. values of PUR and GAS were all less than 10%.

#### 2.4.4. Accuracy

The accuracy (%), i.e., relative recovery, compare the measured concentration with the actual concentration to obtain the method recovery rate, the difference value is the relative recovery rate. The recovery rates of PUR for low, medium and high (0.1, 5, 100 μg·mL^−1^) concentrations were 104.44%, 105.01%, and 100.17%, respectively; the recoveries for low, medium, and high concentrations of GAS were 108.71%, 107.16%, and 99.87%, respectively.

### 2.5. Data Analysis

#### 2.5.1. Transmembrane Resistance

The EVOM cell potentiometer was used to measure the transmembrane resistance of Caco-2 cells, and the transepithelial electrical resistance (TEER) was calculated according to Equation (1).

TEER = (R_1_ − R_0_)·A(Ω·cm^2^) (1)

Before the transport experiment, R_1_ (Ω) is the measured value of the cell group, R_0_ (Ω) is the measured value of the blank group (without inoculated cells); in the transport experiment, R_1_ (Ω) is the measured value of the administered cell group, R_0_ (Ω) is the measured value of the blank cell group (before administration), and A (cm^2^) is the area of the polycarbonate membrane per hole (The area of Transwell membrane used in this experiment is 1.12 cm^2^).

#### 2.5.2. Apparent Permeability Coefficient

The calculation of the apparent permeability coefficients (P_app_) of the Caco-2 cell model refers to the data processing reported by Artursson [30] in 1991 through the P_app_ of the Caco-2 cell monolayer, see Equation (2). The larger the P_app_ value, the higher the permeability.

(2) $$P_{\mathrm{app}}=\frac{\Delta Q}{\Delta t\cdot A\cdot C_{0}}$$

ΔQ is the accumulative transport amount of drug (μg); ΔQ/Δ*t* is the drug transport amount in unit time in the receiving pool, that is, the transport rate (v) (μg·mL^−1^); A is the same as the meaning in the Equation (1); *C*_0_ is the initial mass concentration of the drug in the supply pool (μg·mL^−1^).

The drug efflux ratio (ER) is the ratio of P_app(BL→AP)_ to P_app(AP→BL)_ to the apparent permeability coefficient of the drug transported from the AP side to the BL side.

(3) $$\mathrm{ER}=\frac{P_{\mathrm{app}\left(\mathrm{BL}\to \mathrm{AP}\right)}}{P_{\mathrm{app}\left(\mathrm{AP}\to \mathrm{BL}\right)}}$$

P_app(BL→AP)_ is the P_app_ of drug transported from the BL side to the AP side, and P_app(AP→BL)_ is the P_app_ of drug transported from the AP side to the BL side. It is generally believed that when P_app(BL→AP)_ is close to P_app(AP→BL)_, the drug is absorbed by passive diffusion. When P_app(BL→AP)_/P_app(AP→BL)_ > 1.5, it is indicated that the drug may involve in active transport mechanism.

Since the HBSS blank solution must be supplemented after each sampling, which is equivalent to a dilution effect on the penetration of the drug, the cumulative transport amount (ΔQ) (μg) of the drug can be corrected by the following formula:

(4) $$\Delta Q=C_{n}\cdot V_{R}+V_{S}\cdot \sum_{i=0}^{n-1}C_{i}$$

$C_{n}$ is the permeability concentration of the *n*th sample (μg·mL^−1^); $V_{R}$ is the volume of the receiving cell (mL); $V_{S}$ is the sampling volume at each time point (mL, 0.2 mL in this experiment); $\sum_{i=0}^{n-1}C_{i}$ is the sum of the concentration of samples taken from 0 to n − 1 time points (μg·mL^−1^).

SPSS statistic 17.0 (SPSS Corporation, Chicago, IL, USA) was used for statistical analysis of the data, and the results were expressed as *Mean* ± *SD*.

## 3. Result

### 3.1. Bidirectional Transport of PUR and GAS in Caco-2 Cell Model

#### 3.1.1. Bidirectional Transport of Different Concentrations of PUR

**Figure 3:**
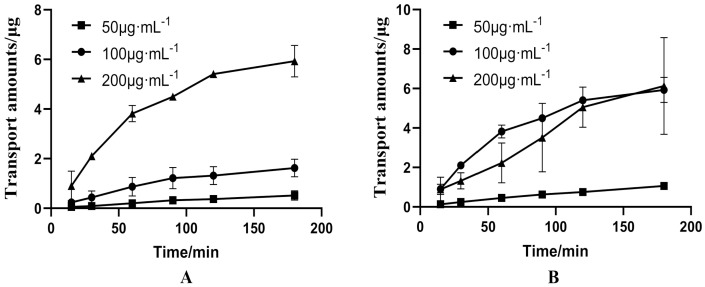
Bidirectional transport amounts of different concentration of puerarin (PUR) in Caco-2 cell model. ((**A**): From AP side to BL side. (**B**): From BL side to AP side).

When the concentration of PUR is 50 μg·mL^−1^, the bidirectional transport amount increases linearly with time, indicating that within this concentration range, the absorption mode of PUR is just passive diffusion (Figure 3). From the AP side to BL side, the transport amount of PUR is concentration-dependent within 180 min, that is, the greater the concentration, the more transported. When the concentration is 100 or 200 μg·mL^−1^, PUR is transported faster within 90 min, and the transport rate tends to be slow after that, indicating that the absorption of PUR is not simply passive absorption within this concentration range, and is protein-mediated active efflux transport mechanism (Figure 3A). From the BL side to AP side, when the concentration of PUR is in the range of 100–200 μg·mL^−1^, the cumulative transport amount of PUR decreases with the concentration increases, indicating that PUR transport is saturated from the BL side to AP side, which also suggests that there may be an active transport mechanism in the absorption of PUR within this concentration range (Figure 3B). Moreover, the ER of 50, 100 and 200 μg·mL^−1^ PUR groups were 1.442 ± 0.073, 3.531 ± 0.129, 2.654 ± 0.693, respectively (Table 2). The ratio of the former group was <1.5, and the ratio of the latter two groups were both >1.5. This also suggested that PUR is passive diffusion at 50 μg·mL^−1^, and there may be an active transport mechanism in the concentration range of 100–200 μg·mL^−1^.

#### 3.1.2. Bidirectional Transport of GAS

**Figure 4:**
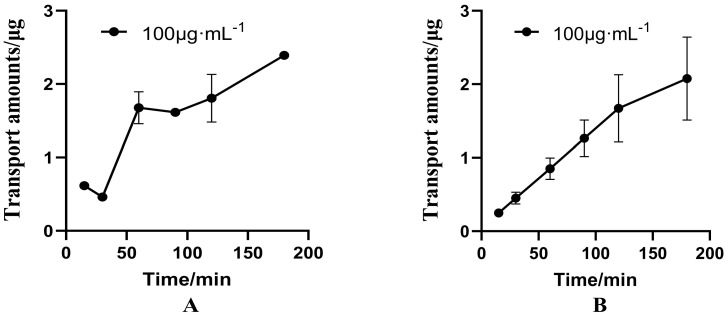
Bidirectional transport amounts of 100 μg·mL^−1^ of gastrodin (GAS) in Caco-2 cell model. ((**A**): From AP side to BL side. (**B**): From BL side to AP side).

This study only investigated the bidirectional transport of GAS at a concentration of 100 μg·mL^−1^ in the Caco-2 cell model (Table 2 and Figure 4). The ER of 100 μg·mL^−1^ GAS was 1.191, which was less than 1.5. In Caco-2 cells, the trans-cell transport of GAS has no obvious directionality, and the transport rate is almost constant.

### 3.2. Effects of Verapamil and Cyclosporin on Transport of PUR and GAS in Caco-2 Cell Model

#### 3.2.1. Effects of Verapamil on Bidirectional Transport of PUR

**Figure 5:**
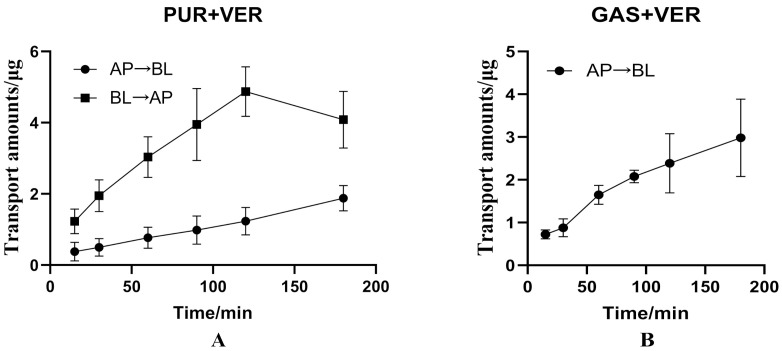

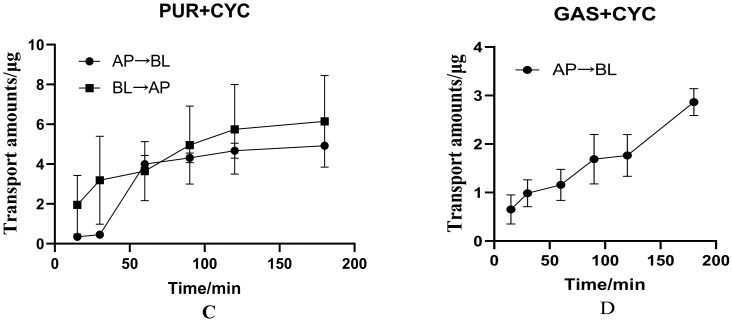
Effects of verapamil and cyclosporin on the transport of puerarin (PUR) or gastrodin (GAS) in Caco-2 cell model. ((**A**): Effects of verapamil on the bidirectional transport of PUR in Caco-2cell model; (**B**): Effects of verapamil on the bidirectional transport of GAS in Caco-2cell model. (**C**): Effects of cyclosporin on the bidirectional transport of PUR in Caco-2cell model. (**D**): Effects of cyclosporin on the bidirectional transport of GAS in Caco-2cell model).

It can be seen from Figure 5A that when 100 μg·mL^−1^ PUR was combined with 100 μmol·L^−1^ verapamil, the ΔQ_(__BL→AP)_ of PUR was reduced, while the ΔQ_(__AP→BL)_ was basically unchanged, that is, the addition of verapamil can reduce the efflux of PUR and finally promote the absorption of PUR. In addition, the absorption of PUR increased (P_app(AP→BL)_ increased from 1.285 to 1.413), the efflux decreased significantly (P_app__(BL→AP)_ decreased from 4.539 × 10^−6^ cm·s^−1^ to 3.004 × 10^−6^ cm·s^−1^), and the ER dropped from 3.531 to 2.126 (a decrease of 39.79%) (Table 3).

#### 3.2.2. Effects of Verapamil on Transport of GAS from AP to BL Side

For GAS, after combined with 100 μmol·L^−1^ verapamil, the ΔQ_(__AP→BL)_ increased, and there was no significant difference in the apparent permeability coefficient between the two groups (Table 3 and Figure 5B).

#### 3.2.3. Effects of Cyclosporin on Bidirectional Transport of PUR

It can be seen from Figure 5C, when 100 μg·mL^−1^ PUR is used in combination with 10 μmol·L^−1^ cyclosporine, the bidirectional cumulative transport amount of the Caco-2 cell monolayer model varies with time. The ΔQ_(__AP→BL)_ of PUR increased, while the ΔQ_(__BL→AP)_ was basically constant in the concentration range of 50–200 μg·mL^−1^. That is, after adding cyclosporin, it appears to increase the absorption of PUR. In additional, the P_app(AP→BL)_ increased significantly from 1.285 × 10^−6^ cm·s^−1^ to 4.759 × 10^−6^ cm·s^−1^, while P_app(BL→AP)_ decreased from 4.539 × 10^−6^ cm·s^−1^ to 4.014 × 10^−6^ cm·s^−1^, a decrease of 76.11% (Table 2 and Table 3).

#### 3.2.4. Effects of Cyclosporin on Transport of GAS from AP to BL Side

For GAS, when combined with 100 μmol·L^−1^ cyclosporine, there is no significant difference in the ΔQ_(__AP→BL)_ and P_app(AP→BL)_ (Table 3 and Figure 5D).

### 3.3. The Interaction between PUR and GAS on Bidirectional Transport in the Caco-2 Cell Model

**Figure 6:**
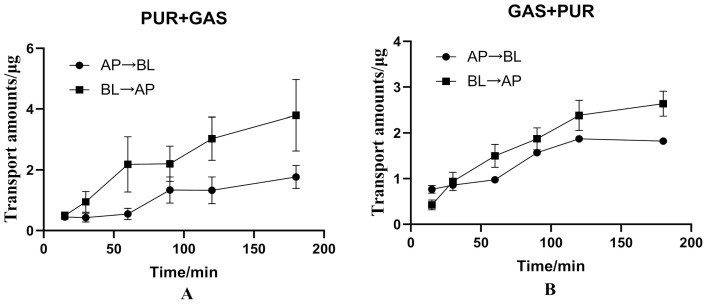
The interaction between puerarin (PUR) or gastrodin (GAS) on bidirectional transport in the Caco-2 cell model. ((**A**): Effects of GAS on the bidirectional transport of PUR in Caco-2cell model. (**B**): Effects of PUR on the bidirectional transport of GAS in Caco-2 cell model).

When 100 μg·mL^−1^ PUR is combined with 100 μg·mL^−1^ GAS, the biodirectional ΔQ were shown in Figure 6. In addition, the ΔQ_(__BL→AP)_ of PUR decreased, while there was no obvious change with the ΔQ_(__AP→BL)_ (Figure 6A). In addition, the P_app(AP→BL)_ of PUR increased (from 1.285 to 1.425, the P_app(BL→AP)_ decreased significantly from 4.539 × 10^−6^ cm·s^−1^ to 3.108 × 10^−6^ cm·s^−1^, and the ER decreased from 3.531 to 2.181, a decrease of 38.22% (Table 4).

As for Gas, when it was combined with PUR, the ΔQ_(__BL→AP)_ of GAS was significant increased, while the ΔQ_(__AP→BL)_ was basically unchanged (Figure 6B). Compared with GAS alone, there was no significant difference in P_app_ value and ER in the combination group. In addition, the ER of 100 μg·mL^−1^ GAS is 1.191, which is less than 1.5 (Table 4).

In summary, the transport of 50 μg·mL^−1^ PUR in Caco-2 cells has no obvious directionality, while the transport of 100 and 200 μg·mL^−1^ PUR presents a strong directionality, and this directionality can be inhibited by verapamil and cyclosporine. The transport of 100 μg·mL^−1^ GAS in Caco-2 cells has no obvious directionality, and the intestinal absorption could not be altered by verapamil and cyclosporine. When PUR and GAS were used in combination, GAS could increase the absorption of PUR while PUR had no obvious influence on GAS.

## 4. Discussion

In the present study, the rationality of the compatibility of PUR and GAS was verified from the perspective of Caco-2 cell transport. It is found that GAS enhanced the P_app(AP→BL)_ significantly, which indicates that GAS can improve the PUR’s intestinal absorption. For intestinal absorption is an important index reflecting the process of drug absorption, so the poor absorption of PUR may closely relate to the intestinal efflux.

P-gp and MRP, its subfamily MRP2, are both expressed in the Caco-2 monolayer cell model, and they can efflux drugs from the inside of the cell to the outside of the cell [31,32]. Verapamil is a P-gp inhibitor, while cyclosporine is both a P-gp inhibitor and an MRP2 inhibitor [33,34]. Therefore, we chose these two inhibitors to elucidate the factors of how GAS enhances the absorption of PUR. Results revealed that the transport of 50 μg·mL^−1^ PUR in Caco-2 cells has no obvious directionality, and the transport rate is basically constant, indicating that the transport of PUR within this concentration range is a passive transport process. The transmembrane transport of 100 and 200 μg·mL^−1^ PUR in Caco-2 cells present a strong directionality, with ER both greater than 1.5, and this tropism can be inhibited by verapamil and cyclosporin. So, it is speculated that PUR is not only a substrate of P-gp, but also a substrate of MRP2. Cyclosporin is more obvious than verapamil to promote transmembrane transport of PUR. Moreover, in addition to passive diffusion, there is also an active transport mode for PUR across the membrane. This result is consistent with the research of Cheng et al. [23]. In addition, it is well known that the efflux function of P-gp is saturated. When the binding sites between P-gp and drugs are almost full, the efflux function decreases significantly [35]. In our study, the ER of 100 and 200 μg·mL^−1^ PUR was 3.531 and 2.654, respectively, which was consistent with the previous research [35]. The transmembrane transport of 100 μg·mL^−1^ GAS in Caco-2 cells has no obvious directionality, and the transport profile could not be altered by verapamil and cyclosporine, suggesting that the transport of GAS is a pure passive process. However, as GAS has no oral malabsorption, thus we just investigated 100 μg·mL^−1^ GAS, did not explore the bidirectional transport of different concentrations of GAS in Caco-2 cells, and the P_app(BL→AP)_ experiment of GAS was also omitted.

When GAS and PUR were used in combination, for PUR, the absorption index P_app(AP→BL)_ increased from 1.285 × 10^−6^ cm·s^−1^ to 1.425 × 10^−6^ cm·s^−1^, the efflux index P_app(BL→AP)_ decreased significantly from 4.539 × 10^−6^ cm·s^−1^ to 3.108 × 10^−6^ cm·s^−1^, and the ER decreased by 38.22%. This is consistent with our previous pharmacokinetic results in rats, which showed that the combined administration of GAS and PUR could increase the absorption, decrease the clearance rate, and prolong the mean retention time, and the bioavailability of the two components when compared with the single-administered group [18]. It is speculated that GAS can play a role similar to verapamil, that is, GAS is a P-gp inhibitor and can promote the transmembrane transport of PUR, which is similar to verapamil. However, whether P-gp and MRP2 have a key influence on the absorption of PUR remains to be further studied.

In this study, the P_app_ values of PUR and GAS were 1.766 × 10^−6^ cm·s^−1^ and 2.866 × 10^−6^ cm·s^−1^, respectively. The P_app_ of PUR is smaller than that of GAS, which is consistent with the determination of the oil-water partition coefficient in our previous study [36]. Studies [37] have shown that, the permeability coefficient of drugs with complete absorption is high (P_app_ > 1 × 10^−6^ cm·s^−1^), and that of drugs with incomplete absorption is low (P_app_ < 1 × 10^−7^ cm·s^−1^) [38,39]. It can be inferred that the two components are well absorbed (not absorbed well) in vivo. Actually, previous studies have shown that PUR is poorly absorbed and GAS is well absorbed [18]. This is because there was a good correlation between oral absorption and P_app_ for passive diffusion drugs. However, for active transport drugs, the P_app_ obtained from Caco-2 cell experiments can only be used as a qualitative rather than quantitative indicator of in vivo absorption.

## 5. Conclusions

The absorption and transport mechanism of PUR and GAS and their interaction were studied and we try to clarify the rationality of their absorption mechanism and compatibility application from the cellular level. The research results show that the compatibility of PUR and GAS is reasonable, and GAS can promote the transmembrane transport of PUR, the effect of which is similar to that of verapamil, which may provide a certain theoretical basis for its combined application.

## Figures and Tables

**Table 1:** Regression data and lower limit of quantitations (LLOQs) of low and high concentration of puerarin (PUR) and gastrodin (GAS) in Caco-2 cell model.

Analyte	Linear Range (μg·mL^−1^)	Linear Regression Equations	Correlation Coefficient (*r*)	LLOQs (μg·mL^−1^)
L-PUR	0.025–2.5	y = 1.8823x + 0.07998	0.9999	0.025
H-PUR	2.5–100	y = 1.8758x − 0.3433	0.9999	2.5
L-GAS	0.1–5	y = 0.2159x + 0.0002	0.9999	0.1
H-GAS	5–100	y = 0.2107x − 0.1703	0.9998	5

L-PUR low dose puerarin; H-PUR high dose puerarin; L-GAS low dose gastrodin; H-GAS high dose gastrodin.

**Table 2:** P_app_ values and efflux ratio (ER) of different concentration of the puerarin (PUR) and gastrodin (GAS) across the Caco-2 monolayer.

Groups	Concentration (μg·mL^−1^)	P_app(AP→BL)_ (×10^−6^ cm·s^−1^)	P_app(BL→AP)_ (×10^−6^ cm·s^−1^)	ER
L-PUR	50	1.225 ± 0.600	1.766 ± 0.090	1.442 ± 0.073
M-PUR	100	1.285 ± 0.332	4.539 ± 0.166	3.531 ± 0.129
H-PUR	200	1.137 ± 0.391	3.017 ± 0.787	2.654 ± 0.693
GAS	100	2.407 ± 0.134	2.866 ± 0.809	1.191 ± 0.336

L-PUR low dose puerarin; M-PUR medium dose puerarin; H-PUR high dose puerarin.

**Table 3:** Effects of verapamil and cyclosporin on P_app_ values and efflux ratio (ER) of puerarin (PUR) or gastrodin (GAS) in Caco-2 cell model.

Groups	Concentration (μg·mL^−1^)	P_app(AP→BL)_ (×10^−6^ cm·s^−1^)	P_app(BL→AP)_ (×10^−6^ cm·s^−1^)	*ER*
PUR + Ver	100	1.413 ± 0.381	3.004 ± 0.724	2.126 ± 0.513
PUR + Cyc	100	4.759 ± 0.405	4.014 ± 0.565	0.843 ± 0.119
GAS + Ver	100	2.647 ± 1.000	—	—
GAS + Cyc	100	2.402 ± 0.130	—	—

Ver verapamil; Cyc cyclosporin.

**Table 4:** P_app_ values and efflux ratio (ER) of the puerarin (PUR) or gastrodin (GAS) across the Caco-2 monolayer.

Groups	Concentration (μg·mL^−1^)	P_app(AP→BL)_ (×10^−6^ cm·s^−1^)	P_app(BL→AP)_ (×10^−6^ cm·s^−1^)	*ER*
PUR + GAS	100	1.425 ± 0.412	3.108 ± 0.982	2.181 ± 0.689
GAS + PUR	100	2.229 ± 0.086	2.566 ± 0.306	1.151 ± 0.137

## Data Availability

Data is contained within the article.

## Sample Availability

Samples of the compounds PUR and GAS are available from the authors.
